# Effects of Eupatilin and Jaceosidin on Cytochrome P450 Enzyme Activities in Human Liver Microsomes

**DOI:** 10.3390/molecules15096466

**Published:** 2010-09-16

**Authors:** Hye Young Ji, Sung Yeon Kim, Dong Kyun Kim, Ji Hyun Jeong, Hye Suk Lee

**Affiliations:** Drug Metabolism & Bioanalysis Laboratory, College of Pharmacy, Wonkwang University, Iksan 570-749, Korea

**Keywords:** eupatilin, jaceosidin, cytochrome P450 inhibition, human liver microsomes, drug-drug interaction

## Abstract

Eupatilin and jaceosidin are bioactive flavones found in the medicinal herbs of the genus *Artemisia*. These bioactive flavones exhibit various antioxidant, antiinflammatory, antiallergic, and antitumor activities. The inhibitory potentials of eupatilin and jaceosidin on the activities of seven major human cytochrome P450 enzymes in human liver microsomes were investigated using a cocktail probe assay. Eupatilin and jaceosidin potently inhibited CYP1A2-catalyzed phenacetin *O*-deethylation with 50% inhibitory concentration (IC_50_) values of 9.4 μM and 5.3 μM, respectively, and CYP2C9-catalyzed diclofenac 4-hydroxylation with IC_50_ values of 4.1 μM and 10.2 μM, respectively. Eupatilin and jaceosidin were also found to moderately inhibit CYP2C19-catalyzed [*S*]-mephenytoin 4'-hydroxylation, CYP2D6-catalyzed bufuralol 1'-hydroxylation, and CYP2C8-catalyzed amodiaquine *N*-deethylation. Kinetic analysis of human liver microsomes showed that eupatilin is a competitive inhibitor of CYP1A2 with a *K_i_* value of 2.3 μM and a mixed-type inhibitor of CYP2C9 with a *K_i_* value of 1.6 μM. Jaceosidin was shown to be a competitive inhibitor of CYP1A2 with a *K_i_* value of 3.8 μM and a mixed-type inhibitor of CYP2C9 with *K_i_* value of 6.4 μM in human liver microsomes. These *in vitro* results suggest that eupatilin and jaceosidin should be further examined for potential pharmacokinetic drug interactions *in vivo* due to inhibition of CYP1A2 and CYP2C9.

## 1. Introduction

Eupatilin [2-(3,4-dimethoxyphenyl)-5,7-dihydroxy-6-methoxychromen-4-one] and jaceosidin [5,7-dihydroxy-2-(4-hydroxy-3-methoxyphenyl)-6-methoxychromen-4-one] are pharmacologically active flavones isolated from medicinal herbs of the genus *Artemisia*, such as *Artemisia princeps, Artemisia argyi*, *Artemisia iwayomogi*, and *Artemisia copa*. Eupatilin and jaceosidin have been shown to exert antioxidant and anti-inflammatory activities [1,2,3,4,5,6,7,8,9] and to possess antiallergic activity [10,11]. Eupatilin and jaceosidin may be used as antimutagens against 3-amino-1-methyl-5*H*-pyrido[4,3-*b*]indole (Trp-P-2), 3-amino-1,4-dimethyl-5*H*-pyrido[4,3-*b*]indole, 2-amino-3-methylimidazo[4,5-*f*]quinoline, 2-amino-3,4-dimethylimidazo[4,5-*f*]quinoline and 2-amino-3,8-dimethylimidazo- [4,5-*f*]quinoxaline in *Salmonella typhimurium* TA98 [12] and as potential cancer chemopreventive agents [13,14,15,16,17,18,19,20,21,22]. Eupatilin exhibits antidiabetic activity by enhancing hepatic glucose metabolism and insulin secretion in type 2 diabetic mice [23]. Eupatilin is extensively metabolized to eupatilin glucuronide, 6-*O*-desmethyl-eupatilin, jaceosidin, and jaceosidin glucuronide in rats [24]. Eupatilin is metabolized to jaceosidin mainly by CYP1A2 in human liver microsomes and eupatilin glucuronidation, a major metabolic pathway, was catalyzed by UGT1A1, UGT1A3, UGT1A7, UGT1A8, UGT1A9, and UGT1A10 [25]. 

The use of botanical drugs to prevent or alleviate common illnesses has increased dramatically in Asian, North American and European countries [26]. Because the botanical drugs share the same metabolic and transport proteins including cytochrome P450 (CYP) enzymes, UDP-glucuronosyltransferase (UGT) enzymes, and drug transporters such as P-glycoprotein with commonly used drugs, the potential for drug-botanical interaction is substantial. FDA guidance has addressed the concern that the interactions between botanical drugs and commonly used drugs and/or dietary supplements should be investigated [27]. The laboratory animal and human studies on drug-interactions of common botanical drugs, including black cohosh, *Echinacea*, garlic, *Ginko biloba*, green tea, kava, milk thistle, and St. John’s wort have been reviewed [28].

To our knowledge, no previous study has reported the effect of eupatilin and jaceosidin on human CYP enzymes. In this study, the effects of eupatilin and jaceosidin on the activities of seven major human CYPs were examined using pooled human liver microsomes to evaluate the possibility of eupatilin and jaceosidin-drug interactions.

## 2. Results and Discussion

The inhibitory effects of eupatilin and jaceosidin toward seven major human CYP isoforms were evaluated by using a cocktail of CYP probe substrates in human liver microsomes. Eupatilin and jaceosidin were found to potently inhibit CYP1A2-dependent phenacetin *O*-deethylation with IC_50_ values of 9.4 μM and 5.3 μM, respectively, and CYP2C9-dependent diclofenac 4-hydroxylation with IC_50_ values of 4.1 μM and 10.2 μM, respectively. Eupatilin and jaceosidin were found to moderately inhibit CYP2C19-catalyzed [*S*]-mephenytoin 4'-hydroxylation with IC_50_ values of 48.1 μM and 64.9 μM, respectively, and CYP2D6-catalyzed bufuralol 1°-hydroxylation (IC_50_ values of 58.7 μM and 79.1 μM, respectively). Eupatilin and jaceosidin were found to weakly inhibit CYP2C8-dependent amodiaquine *N*-deethylation with IC_50_ values of 104.9 μM and 106.4 μM, respectively (Table 1). Eupatilin and jaceosidin showed negligible inhibition of CYP2A6-catalyzed coumarin 7-hydroxylation and CYP3A4-catalyzed midazolam 1'-hydroxylation at the highest concentration tested (100 μM). The inhibitory potencies of eupatilin and jaceosidin were not affected significantly after a 30 min preincubation with microsomes in the presence of NADPH (Table 2), indicating that there is no mechanism-based inhibition. 

**Table 1:** Effects of eupatilin and jaceosidin on CYP metabolic activity in pooled human liver microsomes with IC_50_ values.

CYP activity	CYP	IC_50_ (μM) of eupatilin		IC_50_ (μM) of jaceosidin	
		no preincubation	with preincubation*	no preincubation	with preincubation*
Phenacetin *O*-deethylation	1A2	9.4 ± 1.5	11.8 ± 1.3	5.3 ± 0.81	6.0 ± 0.45
Coumarin 7-hydroxylation	2A6	No inhibition	No inhibition	No inhibition	No inhibition
Amodiaquine *N*-deethylation	2C8	104.9 ± 7.2	110.1 ± 6.5	106.4 ± 8.6	135.5 ± 9.4
Diclofenac 4-hydroxylation	2C9	4.1 ± 0.49	4.9 ± 0.91	10.2 ± 1.0	8.6 ± 0.91
*S*-Mephenytoin 4'-hydroxylation	2C19	48.1 ± 12.0	57.7 ± 16.3	64.9 ± 4.8	69.8 ± 3.8
Bufuralol 1'-hydroxylation	2D6	58.7 ± 7.6	79.1 ± 7.5	79.1 ± 1.8	82.3 ± 2.4
Midazolam 1'-hydroxylation	3A	No inhibition	No inhibition	No inhibition	No inhibition

*Eupatilin and jaceosidin were preincubated for 30 min in the presence of NADPH before the addition of the substrate. Cocktail substrate concentrations used for the assessment of IC_50_ were as follows: 50 μM phenacetin, 2.5 μM coumarin, 2.0 μM amodiaquine, 10 μM diclofenac, 50 μM [*S*]-mephenytoin, 5.0 μM bufuralol, and 2.5 μM midazolam.

**Table 2:** *K*_i_ values for the inhibition of CYP1A2, CYP2C8, CYP2C9, CYP2C19, and CYP2D6 activities by eupatilin and jaceosidin in pooled human liver microsomes.

CYP	Marker reactions	*K*_i _ (μM)	
		eupatilin	jaceosidin
1A2	Phenacetin *O*-deethylation	2.3 ± 0.18 (competitive)	3.8 ± 0.23 (competitive)
2C8	Amodiaquine *N*-deethylation	101.9 ± 8.9 (competitive)	109.4 ± 7.3 (competitive)
2C9	Diclofenac 4-hydroxylation	1.6 ± 0.17 (mixed, α^*^ = 10.3)	6.4 ± 0.25 (competitive)
2C19	*S*-Mephenytoin 4'-hydroxylation	28.7 ± 3.6 (mixed, *α* = 8.7)	45.1 ± 4.2 (mixed, *α* = 3.7)
2D6	Bufuralol 1'-hydroxylation	94.6 ± 5.8 (competitive)	57.8 ± 3.6 (competitive)

*α: The factor by which *Km* changes when an inhibitor occupies the enzyme.

**Figure 1:**
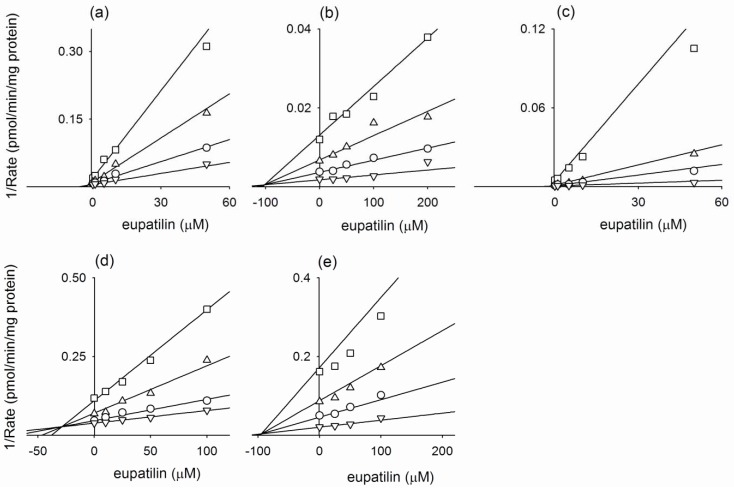
Representative Dixon plots for inhibitory effects of eupatilin on (a) CYP1A2-catalyzed phenacetin *O*-deethylation, (b) CYP2C8-catalyzed amodiaquine *N*-deethylation, (c) CYP2C9-catalyzed diclofenac 4-hydroxylation, (d) CYP2C19-catalyzed [*S*]-mephenytoin 4'-hydroxylation, and (e) CYP2D6-catalyzed bufuralol 1'-hydroxylation in pooled human liver microsomes. Each symbol represents the substrate concentration. (a) phenacetin: 10 μM (▽), 20 μM (○), 40 μM (△), and 80 μM (□); (b) amodiaquine:0.5 μM (▽), 1.0 μM (○), 2.0 μM (△), and 5.0 μM (□); (c) diclofenac: 1 μM (▽), 5 μM (○),10 μM (△), and 50 μM (□); (d) [*S*]-mephenytoin: 10 μM (▽), 20 μM (○), 50 μM (△), and 100 μM (□); (e) bufuralol: 0.5 μM (▽), 1.0 μM (○), 2.0 μM (△), and 5.0 μM (□). Each data point represents the mean of triplicate experiments.

**Figure 2:**
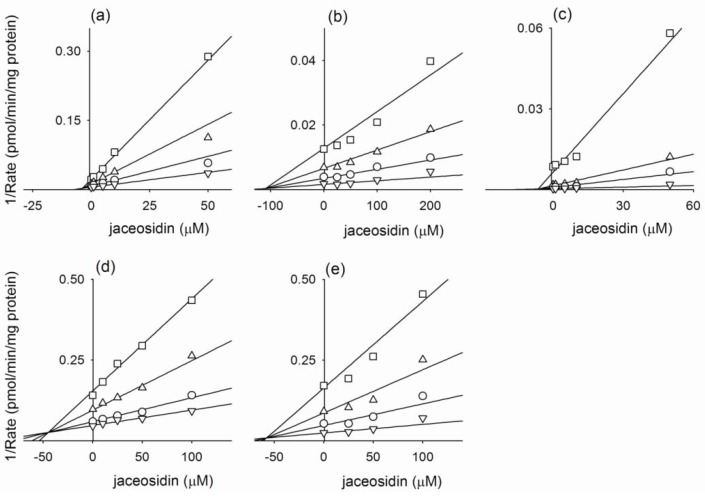
Representative Dixon plots for inhibitory effects of jaceosidin on (a) CYP1A2-catalyzed phenacetin *O*-deethylation, (b) CYP2C8-catalyzed amodiaquine *N*-deethylation, (c) CYP2C9-catalyzed diclofenac 4-hydroxylation, (d) CYP2C19-catalyzed [*S*]-mephenytoin 4'-hydroxylation, and (e) CYP2D6-catalyzed bufuralol 1'-hydroxylation in pooled human liver microsomes. Each symbol represents the substrate concentration. (a) phenacetin: 10 μM (▽), 20 μM (○), 40 μM (△), and 80 μM (□); (b) amodiaquine:0.5 μM (▽), 1.0 μM (○), 2.0 μM (△), and 5.0 μM (□); (c) diclofenac: 1 μM (▽), 5 μM (○),10 μM (△), and 50 μM (□); (d) [*S*]-mephenytoin: 10 μM (▽), 20 μM (○), 50 μM (△), and 100 μM (□); (e) bufuralol: 0.5 μM (▽), 1.0 μM (○), 2.0 μM (△), and 5.0 μM (□). Each data point represents the mean of triplicate experiments.

In an inhibition study, it was found that the apparent *K*_i_ value is a more appropriate parameter for defining the interaction of the inhibitor with a particular enzyme. The *K*_i_ values and inhibition types (competitive, noncompetitive, uncompetitive, or mixed) were determined for eupatilin and jaceosidin using Lineweaver plots, Dixon plots, and secondary reciprocal plots. The results are summarized in Table 2. Eupatilin showed the mixed-type inhibition for CYP2C9-catalyzed diclofenac 4-hydroxylation (*K*_i_, 1.6 μM; *α*, 10.3) and CYP2C19-catalyzed [*S*]-mephenytoin 4'-hydroxylation (*K*_i_, 28.7 μM; *α*, 8.7) (Figure 1, Table 2). Eupatilin was found to competitively inhibit CYP1A2-catalyzed phenacetin *O*-deethylation (*K*_i_, 2.3 μM), CYP2C8-dependent amodiaquine *N*-deethylation (*K*_i_, 101.9 μM), and CYP2D6-catalyzed bufuralol 1'-hydroxylation (*K*_i_, 94.6 μM) (Figure 1, Table 2). Jaceosidin was found to exhibit competitive inhibition for CYP1A2-catalyzed phenacetin *O*-deethylation (*K*_i_, 3.8 μM), CYP2C8-dependent amodiaquine *N*-deethylation (*K*_i_, 109.4 μM), CYP2C9-catalyzed diclofenac 4-hydroxylation (*K*_i_, 6.4 μM), and CYP2D6-catalyzed bufuralol 1'-hydroxylation (*K*_i_, 57.8 μM). Jaceosidin was found to exhibit the mixed type of inhibition for CYP2C19-catalyzed [*S*]-mephenytoin 4'-hydroxylation with a *K*_i_ of 45.1 μM (Figure 2, Table 2). 

Eupatilin and jaceosidin were found to be potent competitive inhibitors of CYP1A2 with *K*_i_ values of 2.3 and 3.8 μM, respectively, indicating that eupatilin and jaceosidin should be used carefully with drugs metabolized by CYP1A2 such as acetaminophen, caffeine, phenacetin, ropivacaine, tacrine, theophylline, and tizanidine to avoid drug interactions [29]. Some natural compounds including oltipraz, trans-resveratrol, tanshinone I, tanshinone IIA, and cryptotanshinone have been shown to potently inhibit CYP1A2 [29,30]. These results support the report that the antimutagenic activity of eupatilin and jaceosidin against Trp-P-2 in TA98 is a result of the inhibition of CYP1A1-catalyzed ethoxyresorufin deethylase activity in rat liver S9 fractions [12]. Eupatilin and jaceosidin were shown to be potent competitive inhibitors of CYP2C9 with *K*_i_ values of 1.6 and 6.4 μM, respectively, suggesting that eupatilin and jaceosidin should be used carefully with drugs metabolized by CYP2C9 such as celecoxib, diclofenac, glyburide, losartan, tolbutamide, torasemide, and *S*-warfarin to avoid drug interactions [31]. The herbal preparations and medicinal herbs containing eupatilin and jaceosidin may affect CYP1A2 and CYP2C9 activity. These *in vitro* results suggest that eupatilin and jaceosidin should be examined for potential pharmacokinetic drug interactions *in vivo* due to inhibition of CYP1A2 and CYP2C9.

## 3. Experimental

### 3.1. Materials and reagents

Eupatilin and jaceosidin were donated by Dong-A Pharmaceutical Co. (Yongin, Korea) and the Plant Diversity Research Center, Korea Research Institute of Bioscience and Biotechnology (Daejeon, Korea), respectively. NADPH, phenacetin, acetaminophen, coumarin, 7-hydroxycoumarin, diclofenac and midazolam were purchased from Sigma Chemical Co. (St. Louis, MO, USA). Pooled human liver microsomes (H161), bufuralol, [*S*]-mephenytoin, 4-hydroxydiclofenac, 4'-hydroxymephenytoin, 1'-hydroxybufuralol, *N*-desethylamodiaquine, 1'-hydroxymidazolam, [^2^H_9_]-1'-hydoxybufuralol maleate, [^2^H_5_]-7-hydroxycoumarin, [^2^H_3_]-4'-hydoxymephenytoin, [^13^C_2_,^15^N]-acetaminophen, and [^13^C_6_]-4-hydoxydiclofenac were obtained from BD Gentest Co. (Woburn, MA, USA). Acetonitrile and methanol (HPLC grade) were obtained from Burdick & Jackson Inc. (Muskegon, MI, USA) and the other chemicals were of the highest quality available. 

### 3.2. Inhibitory effects of eupatilin and jaceosidin on 7 major CYP activities in human liver microsomes

The inhibitory potencies (IC_50_ values) of eupatilin and jaceosidin were determined with CYP assays in the presence and absence of eupatilin and jaceosidin (final concentrations of 0.1−100 μM with acetonitrile concentration of less than 0.5% v/v) using pooled human liver microsomes. Phenacetin *O*-deethylase, coumarin 7-hydroxylase, amodiaquine *N*-deethylase, diclofenac hydroxylase, [*S*]-mephenytoin 4'-hydroxylase, bufuralol 1'-hydroxylase, and midazolam 1'-hydroxylase activities were determined as probe activities for CYP1A2, CYP2A6, CYP2C8, CYP2C9, CYP2C19, CYP2D6, and CYP3A, respectively, using cocktail incubation and tandem mass spectrometry. The incubation mixtures were prepared in a total volume of 200 μL as follows: pooled human liver microsomes (0.25 mg/mL), 1.3 mM NADPH, 3.3 mM MgCl_2_, 50 mM potassium phosphate buffer (pH 7.4), and a cocktail of probe substrates and various concentrations of eupatilin or jaceosidin (0.1−100 μM). The substrates were used at concentrations approximately equal to their respective *K*_m_ values: 50 μM phenacetin, 2.5 μM coumarin, 2 μM amodiaquine, 10 μM diclofenac, 100 μM [*S*]-mephenytoin, 5 μM bufuralol, and 2.5 μM midazolam. After a 3 min preincubation at 37 ºC, the reactions were initiated by adding NADPH and incubated for 20 min at 37 ºC with shaking in a water bath. After incubation, the reaction was stopped by placing the tubes on ice and adding 100 μL of ice-cold acetonitrile containing internal standards (0.5 μg/mL [^13^C,^15^N]-acetaminophen for acetaminophen and *N*-desethylamodiaquine, 0.5 μg/mL [^2^H_5_]-7-hydroxycoumarin for 7-hydroxycoumarin, 2.0 μg/mL [^13^C_6_]-4-hydoxydiclofenac for 4-hydoxydiclofenac, 0.5 μg/mL [^2^H_3_]-4'-hydoxymephenytoin for 4-hydoxymephenytoin and 1'-hydroxymidazolam, and 0.5 μg/mL [^2^H_7_]-1'-hydroxybufuralol for 1'-hydroxybufuralol). The incubation mixtures were then centrifuged at 10,000× g for 5 min. All incubations were performedin triplicate. 

To evaluate the effect of preincubation on inhibitory potency, eupatilin and jaceosidin (0.1−100 μM) were preincubated for 30 min with NADPH, buffer, and microsomes before the addition of the CYP probe substrates. The reaction was started by addition of the cocktail of probe substrates. 

### 3.3. Kinetic analysis

For the determination of *K*_i _values, human liver microsomes (0.2 mg/mL) were incubated with various concentrations of substrates (10−100 μM phenacetin for CYP1A2, 0.5−5 μM amodiaquine for CYP2C8, 1−50 μM diclofenac for CYP2C9, 10−100 μM [*S*]-mephenytoin for CYP2C19, and 0.5−5 μM bufuralol for CYP2D6), 1 mM NADPH, 10 mM MgCl_2_, and various concentrations of jaceosidin or eupatilin in 50 mM potassium phosphate buffer (pH 7.4) in a total incubation volume of 200 μL. The reactions were initiated by addition of NADPH at 37 ºC and stopped after 15 min by placing the incubation tubes on ice and adding 100 μL of ice-cold methanol containing an internal standard. The incubation mixtures were centrifuged at 10,000' g for 5 min, and aliquots (5 μL) of the supernatants were analyzed by liquid chromatography/tandem mass spectrometry (LC/MS/MS).

### 3.4. LC/MS/MS analysis

All seven metabolites produced from the cocktail of CYP isoform-specific substrates were simultaneously determined by LC/MS/MS. The system consisted of a tandem quadrupole mass spectrometer (TSQ Quantum Access, ThermoFisher Scientific, CA, USA) coupled with a Nanospace SI-2 LC system. The separation was performed on an Atlantis dC_18_ column (5 μm, 2.1 mm i.d. × 100 mm, Waters, Waltham, MA, USA) using the gradient elution of a mixture of 5% methanol in 0.1% formic acid (mobile phase A) and 95% methanol in 0.1% formic acid (mobile phase B) at a flow rate of 0.25 mL/min: 10% mobile phase B for 1 min, 10% to 95% mobile phase B for 1 min, 95% mobile phase B for 5 min. The column and autosampler temperatures were 50 ºC and 4 ºC, respectively. After 1.5 min, the LC eluant was diverted from waste to the mass spectrometer that was fitted with an electrospray ionization (ESI) source and operated in the positive ion mode. The ESI source settings for the ionization of the metabolites were as follows: electrospray voltage, 5.0 kV; vaporizer temperature, 420 ºC; capillary temperature 360 ºC; sheath gas pressure, 35 psi; auxiliary gas pressure, 10 psi. Quantification was performed by selected reaction monitoring (SRM) of the [M+H]^+^ ion and the related product ion for each metabolite. SRM transitions for the metabolites and internal standards are summarized in Table 3. The analytical data were processed using Xcalibur^® ^software (ThermoFisher Scientific).

**Table 3:** LC/MS/MS measurement conditions for drug oxidation catalyzed by human CYP.

CYP	Compound		SRM Transitions	Tube lens (V)	Collision energy (V)
CYP1A2	Metabolite	acetaminophen	152.19>110.19	59	23
	Internal standard	[^13^C_2_,^15^N]-acetaminophen	155.05>111.29	58	21
CYP2A6	Metabolite	7-hydroxycoumarin	163.04>107.38	70	22
	Internal standard	[^2^H_5_]-7-hydroxycoumarin	168.00>112.53	73	22
CYP2C8	Metabolite	*N*-desethylamodiaquine	328.01>282.64	45	19
	Internal standard	[^13^C_2_,^15^N]-acetaminophen	155.05>111.29	58	21
CYP2C9	Metabolite	4-hydroxydiclofenac	312.12>231.05	54	23
	Internal standard	[^13^C_6_]-4-hydroxydiclofenac	318.49>237.28	54	20
CYP2C19	Metabolite	4'-hydoxymephenytoin	235.03>150.19	50	27
	Internal standard	[^2^H_3_]-4'-hydoxymephenytoin	238.18>150.40	50	25
CYP2D6	Metabolite	1'-hydroxybufuralol	278.08>186.31	54	19
	Internal standard	[^2^H_9_]-1'-hydroxybufuralol	287.12>187.09	54	20
CYP3A	Metabolite	1'-hydroxymidazolam	342.08>324.09	73	25
	Internal standard	[^2^H_3_]-4'-hydoxymephenytoin	238.18>150.40	50	25

### 3.5. Data analysis

The IC_50_ values (concentration of inhibitor causing 50% inhibition of the original enzyme activity) were calculated using WinNonlin software, a non-linear regression analysis program (Pharsight, Mountain View, CA, USA). The apparent kinetic parameters for inhibitory potential (*K*_i_ values) were estimated from the fitted curves using Enzyme Kinetics Ver. 1.3 program (Systat Software Inc., San Jose, CA, USA).

## 4. Conclusions

By screening the inhibitory effects of eupatilin and jaceosidin on the activities of seven CYP isoforms in human liver microsomes, eupatilin and jaceosidin were shown to be the potent inhibitors of CYP1A2 and CYP2C9 and moderate inhibitors of CYP2C8, CYP2C19, and CYP2D6. Eupatilin and jaceosidin are pharmacologically active components in medicinal herbs such as *Artemisia *spp. as well as *Eupatorium* spp. These results suggest that the use of eupatilin, jaceosidin, and herbal preparations thereof may cause interactions with drugs metabolized by CYP1A2 and CYP2C9 in some individuals. However, it is notable that *in vitro* inhibition of CYP1A2 and CYP2C9 activities does not necessarily result in drug interactions in clinical situations.
